# P-2085. Optimizing GAIHN-AR Network Microbiology Laboratory Assets for Early Detection of Carbapenemase-Producing Organisms in Limited Resources Settings: Argentine Experience

**DOI:** 10.1093/ofid/ofae631.2241

**Published:** 2025-01-29

**Authors:** Alejandra Corso, Fernando Pasteran, Paulina Marchetti, Andrea Appendino, Rosana Pereda, Alejandra Menocal, Ana Sangoy, Marilina Kuzawka, Eugenia Tocho, Antonela Cioffi, Juan Manuel de Mendieta, Celeste Lucero, Angel M Colque, Laura Alonso, Paula Iantorno, Irene Pagano, Laura Barcelona

**Affiliations:** ANLIS-Malbrán, Buenos Aires, Ciudad Autonoma de Buenos Aires, Argentina; ANLIS-Malbrán, Buenos Aires, Ciudad Autonoma de Buenos Aires, Argentina; INEI ANLIS Dr. C. G. Malbran, Ciudad Autonoma de Buenos Aires, Ciudad Autonoma de Buenos Aires, Argentina; Houssay Hospital, Buenos Aires, Buenos Aires, Argentina; Hospital de Niños P. de Elizalde, Buenos Aires, Buenos Aires, Argentina; INEI ANLIS Dr. C. G. Malbran, Ciudad Autonoma de Buenos Aires, Ciudad Autonoma de Buenos Aires, Argentina; Houssay Hospital, Buenos Aires, Buenos Aires, Argentina; Elizalde Hospital, Buenos Aires, Ciudad Autonoma de Buenos Aires, Argentina; Houssay Hospital, Buenos Aires, Buenos Aires, Argentina; Elizalde Hospital, Buenos Aires, Ciudad Autonoma de Buenos Aires, Argentina; INEI ANLIS Dr. C. G. Malbran, Ciudad Autonoma de Buenos Aires, Ciudad Autonoma de Buenos Aires, Argentina; INEI ANLIS Dr. C. G. Malbran, Ciudad Autonoma de Buenos Aires, Ciudad Autonoma de Buenos Aires, Argentina; Complejo Medico Churruca Visca, Ciudad Autonoma de Buenos Aires, Ciudad Autonoma de Buenos Aires, Argentina; Instituto Nacional de Epidemiologia “Dr Juan H. Jara” Anlis Malbran, Buenos Aires, Ciudad Autonoma de Buenos Aires, Argentina; Comisión Nacional de Resistencia a los Antimicrobianos, Buenos Aires, Ciudad Autonoma de Buenos Aires, Argentina; INE ANLIS Dr. C. G. Malbran, Mar del Plata, Buenos Aires, Argentina; Comisión Nacional de Resistencia a los Antimicrobianos, Buenos Aires, Ciudad Autonoma de Buenos Aires, Argentina

## Abstract

**Background:**

The Global Action in Healthcare Network-Antimicrobial Resistance Module (GAIHN-AR), led by the U.S. CDC, enhances prevention, detection and response in low-resource hospital settings (LRS) against emerging antimicrobial resistance (AR) threats, with an initial focus on carbapenemase-producing organisms. GAIHN-AR includes a lab. component for improving early AR detection, an infection prevention and control program, and a communication platform for rapid response coordination. We describe the implementation verification/validation of new AR diagnostic methods and their integration into clinical workflow

Table

**Figure:**
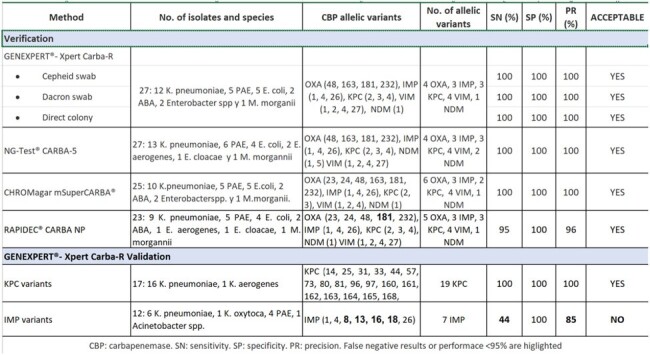

**Methods:**

Prior to GAIHN-AR initiation in two hospitals in Argentina in January 2023, validation of CPO diagnostic techniques was conducted. For this, reference strains characterized by WGS from the CDC & FDA AR-Bank and Argentina-NRL repository were used. Evaluation followed manufacturer specifications, with GeneXpert also tested with alternative Dacron swabs. Pure colonies were used for validation of all methods, except for GENEXPERT®Carba-R where contrived fecal sample were also used. Methodologies were acceptable with sensitivity, specificity, and precision values ≥ 95%.The following methodologies were verified for carbapenemase detection in *Enterobacterales*, *P. aeruginosa*, and *Acinetobacter* spp. (ACI): (i) GENEXPERT®Carba-R; (ii) NG-Test®CARBA-5 lateral flow (ACI excluded); (iii) CHROMagar mSuperCARBA® chromogenic medium for carbapenem resistance detection; (iv) RAPIDEC® CARBA-NP. Additionally, the GENEXPERT®Carba-R was validated for: i) KPC variants with ceftazidime/avibactam resistance, ii) local circulation metallo-β-lactamase IMP variants

**Results:**

The methodologies under evaluation achieved performance between 95-100%, except for the IMP variants with GenXpert Carba-R where sensitivity was reduced to 44% (Table).

**Conclusion:**

Verification/validation process demonstrated that most methods had acceptable performance, allowing integration into lab workflows to facilitate prompt diagnosis and rapid communication with IPC teams. Results benefited not only GAIHN-AR hospitals but also other LRS facilities with whom they were shared through the NRL, offering valuable lessons for similar settings

**Disclosures:**

All Authors: No reported disclosures

